# Predictors of birth weight in pregnant women with malaria: a prospective cohort facility-based study in Webuye-Kenya

**DOI:** 10.1186/s12884-024-06355-9

**Published:** 2024-03-08

**Authors:** Joseph Mukala, Dominic Mogere, Peter Kirira, Bernard N. Kanoi, Violet Akisa, Francis Kobia, Harrison Waweru, Jesse Gitaka

**Affiliations:** 1 School of Public Health, Mount Kenya University, P.O. Box 342-01000, Thika, Kenya; 2 School of Applied Sciences, Mount Kenya University, Thika, Kenya; 3 Directorate of Research and Innovation, Mount Kenya University, Thika, Kenya; 4 Centre for Malaria Elimination, Mount Kenya University, Thika, Kenya; 5 Webuye County Hospital, Webuye, Kenya

**Keywords:** Predictors, Birthweight, Malaria, Pregnant women

## Abstract

In sub-Saharan Africa, malaria, which remains a major public health burden, has a prevalence of 9 to 28% and malaria in pregnancy is associated with severe adverse outcomes for the mother and her baby. Here, we sought to determine the predictors of birth weight in a cohort of 140 women with malaria in pregnancy, who were recruited at the Webuye County hospital in Western Kenya. All study participants underwent malaria diagnosis through microscopic examination of blood smear samples and were grouped into the malaria-positive and malaria-negative groups. Both groups were followed up beginning at the first antenatal visit (March 2022) until delivery (December 2022) and various data, including demographic, parity, gravidity, socioeconomic, maternal and fetal outcomes were collected. Data analyses were done using SPSS version 27. Chi-square and Fisher’s Exact tests were used for bivariate and relative risk analyses at a *p*-value of ≤0.05 (95%) confidence level. Most of the participants were aged 18–25 years, were primigravidas and married, had secondary school-level education, earned 20﻿–30 thousand Kenya shillings, resided in rural areas, and were in the second trimester. There were 6 (4.6%) cases of low birth weight, 3 (4.5%) in the malaria-negative group and 3 (4.7%) in the malaria-positive group. During pregnancy, 41 (31.5%) were anaemic, 5 (3.8%) were HIV-positive, 5 (3.8%) had preeclampsia, and 2 (1.5%) had gestational diabetes. Our analyses show that confounding factors like anaemia, HIV, pre-eclampsia and gestational diabetes did not influence birthweight (*p *≥ 0.923). The malaria-positive and malaria-negative groups did not differ significantly with regard to the low birth weight (relative risk: 0.999, 95% confidence interval: 0.926–1.077). Marital status, gestational age, and area of residence were associated with malaria *p *≤ 0.001, ≤ 0.001 and 0.028 respectively. In both groups, 124 of the ﻿140 deliveries had normal birth weights and of these 63 (95.4%, *n *=﻿ 70) were in the malaria-negative group, whereas 61 (95.3%, *n *= 70) belonged to the malaria-positive group.

## Introduction

Globally malaria affects approximately 515 million people in Latin America, Asia and Sub-Saharan Africa region with one to three million deaths each year [1]. Recently, malaria has affected 228 million people worldwide, with approximately 213 million in the Sub-Saharan Africa either 93% of the world population [2]. Statistics show that 9.6 million people either 19% of the total population in Kenya were at risk of malaria in 2019 in the high altitude or highland zones. Bungoma, Kakamega and Baringo are located in this region, and they are also endemic zones along the Lake Victoria. The coastal region had 13.7 million representing 27% of population at risk of malaria. The seasonal malaria transmission zone is in northern and central Kenya; the number of exposed persons were 11 million representing 23% of the population at risk of malaria, while Nairobi being the capital city of Kenya accounts for 15 million representing 35% of population with low malaria risk [3]. In the central Africa region pregnancy exposed malaria cases were estimated at 3.4 million with multiple consequences ranging from deadly complications such as low birth weight, anemia, abortion, intrauterine fetal retardation, small gestational for age, prematurity. However, many cases of low birth weight were averted via the use of intermittent preventive treatment chemoprevention [1]. Worldwide, low birth weight represents 15 to 20%, while in 2022, there were 35.4 million pregnancies, of which 12.7 million (36%) were exposed to malaria hence without specific prevention, malaria would have resulted in 914,000 newborns with low birthweight [2]. Among as many as eleven million pregnant women who were exposed to malaria infection in 2018, the consequences translated to an estimated 872,000 low birth weights newborns being the highest record of 16% in the Western Africa region compared to the central and Eastern Africa [4]. Interestingly, countries that recorded less than hundred cases of malaria among autochthones population increased from 17 in 2010, to 25 in 2017, and finally 27 in 2018. Moreover, Algeria and Malaysia have not so far reported cases of malaria while China has been awarded a certificate of malaria elimination by [5, 6]. The Kenya National Malaria Program working closely in partnership with other supportive agencies to assist the districts and counties as the execution level of ensuring the smooth process in line with prevention, detection, management of malaria cases based on WHO recommendations [7]. In a research study conducted in the Democratic Republic of Congo, it was highlighted that socio-demographic factors like marital status was associated with malaria based on whether the participant was single or married, while another study demonstrated the association between malaria and gestational age. However, the findings did not explain the statistical association between different trimesters and malaria [8, 9]. *Plasmodium falciparum* infects red blood cells and infected blood cells in the placenta cause sequestration in the intervillous spaces with consequences free radicals release of cytokines and other proteins inducing inflammatory, and immune response leading to oxidative stress, placental hypoperfusion and cells death due to excessive immune response leading to various complications such as fetal growth restriction, stillbirth, low birth weight and prematurity [10]. Malaria in pregnancy study carried out in Bungoma County found a prevalence of 21.6% with high likelihood of infection during the first trimester of gestation when compared with other trimesters. This study found that the prevalence of malaria in second trimester was 66%. Therefore, supporting the recommendation of WHO to increase the effort to curb the high prevalence of malaria among pregnant women living in endemic zones [11]. The low birth weight could be further controlled with effective strategies which can control risk factors associated with malaria in pregnancy such as marital status, gestational age and geographical location or area of residence.

### Problem statement

Globally, malaria remains a public health burden due to its high prevalence and multiple consequences for both women and their newborns. In 2022, there were 228 million people affected of whom 213 million in the Sub-Saharan Africa region either 93% of the world population, and 34.4 million pregnancies of which 12.7 million were exposed to malaria [1]. The prevalence of malaria has remained relatively higher and varies in different transmission zones with 27% in Nigeria, 12% in the Democratic Republic of Congo, 5% in Uganda and 4% Mozambique [1]. In Kenya, the prevalence varies between 9 to 18% even 28% among pregnant women living along the Victoria lakes with high morbidity and mortality backed by factors such as socio-demographic, geographical location, level of acquired immunity and individual transmission intensity [12]. Low birth weight is defined as a birth weight less than 2500 g and is the most common malaria complication representing 10–20% of newborns affected as a result of malaria associated with neonatal morbidity and mortality [13]. A previous study demonstrated that the utilization and the uptake of highly cost-effective interventions such as sulfadoxine-pyrimethamine and mosquito treated nets are associated with poor maternal knowledge, complicated guidelines and policies issues baring healthcare workers to deliver efficient routine antenatal care [14]. The magnitude of low birth weight as a result of malaria during pregnancy need to be established in Bungoma County as one of prone malaria zones. This is the reason as to why this research was being carried out to determine predictors of birth weight in pregnant women with malaria in a cohort study in Webuye hospital.

### Justification

In malaria endemic settings like in Bungoma/Kenya, prevalence of malaria is still elevated among pregnant women and children. Exposure to *Plasmodium falciparum* has been associated with low birth weight and other critical consequences despite effective preventive measures to curb the trends of malaria during pregnancy. Low birth weight represents a high risk for neonatal morbidity and mortality. However, there are no current studies conducted in Bungoma which could inform the magnitude of low birth weight as a result of malaria exposure during pregnancy and the associated risk factors.

### Significance

In the era dedicated to malaria elimination, information related to low birth weight specifically due to malaria among pregnant women still are needed in endemic malaria zones for the rationalization of preventive and curative interventions. Such information could contribute to better decision making regarding the enhancement of control and preventive interventions against malaria, and at the same time can be used at both higher and community level for a collective strategy to promote good practices within and outside the surrounding Counties.

## Materials and methods

### Study design

The study design was a prospective cohort conducted at Webuye hospital from (March 2022) to (December 2022), either 10 months. The tool response rate was 97% with 140 participants out of 144 who returned the questionnaire. Enrolled participants were from 16 weeks gestation and further subdivided in two arms; malaria positive and malaria negative, thereafter followed until delivery.

### Study setting

The study was conducted in Bungoma County Code 39, Sub-County Webuye West code 3911, Webuye hospital. The County has a population estimated at 1,919,490 with 939,105 males and 980,385 females, 429,762 women of childbearing aged between 15 and 49 years. There are 12 sub-Counties, 45 wards and 149 Sub-locations. The County covers an area of 3032 km^2^ and lies between a latitude 00 28’and latitude 10 30′ North of the Equator, and longitudinal 340 20′East and 350 15′East of the Greenwich meridian [15]. It borders the Republic of Uganda to the Northwest, Trans-Nzoia County to North-East, Kakamega County to the East and South-East, and Busia County to the West and Southwest with two rainy seasons: a long from March to July and a short from August to October, an annual rainfall of 400 mm to 1800 mm as well as a temperature between 0 °C and 32 °C [15].

### Sampling frame and inclusion criteria

The expected population was 144 subjects to be enrolled systematically in either arm 1 if malaria turned positive or arm 2 if malaria was negative. A total of 140 pregnant women aged between 18 and 49 years with gestation from 16 weeks were obtained and enrolled prospectively at the antenatal clinic. The participants must have been living in the area for almost 6 months. Microscopy test was used to diagnose malaria, whereby out of the 140 participants enrolled, 70 (50%) tested positive and 70 (50%) tested negative.

### Data collection

Data were collected using the following steps; questionnaires were adapted from WHO and UNICEF antenatal care model. Assistant researchers were trained and some questions were corrected and reformulated. Pretesting of questionnaires was done in mother child health service Webuye hospital, where 15–20 pre-selected pregnant women were administered the questions 2 weeks before the beginning of the study. Thereafter, the corrected version was adopted in English and Kiswahili with supervisor’s approval. Socio-demographics variables were: age groups (18–25, 26–33, 34–41, 42–49), gestation (first:1–12, second:13–25, third:26–38 weeks), education level (none, primary, secondary, college/university), income earning (low:10-20Ksh, middle:21-35Ksh, high: >35Ksh), marital status (married, divorced, single, widowed, monogamous, polygamous), residence (rural, urban), occupation (housewife, employed, self-employed), residence (rural, urban), distance to the health facility (in hour or minutes). The outcome variables: low birth weight (< 2500 g), normal birth weight (> 2500), normal delivery (alive newborn). The following registers were used: MOH ANC (405), Maternity register (333), laboratory register (204).

## Detection and quantification of malaria parasites

### Microscopy

The lobe of finger was disinfected and a pricking obtained as well as a large drop of blood collected on the microscope slide. Thick smear obtained via spreading of the drop on a transparent area of 1 cm^2^, air-dry the slide in horizontal position followed by slide staining with Giemsa 10% and 90 mls of buffered water for approximately 30 minutes. Slides were examined microscopically using 100 magnifications, trophozoites/schizonts were identified over a parasitemia density measured in 1 microliter of blood which was calculated through quantification of malaria parasites number versus 200 WBC multiplied to 8000 to determine parasitemia one blood microliter. After 100 high-power fields visualization the results were either negative if no parasites found or positive if malaria parasites seen. Therefore, quality control was achieved through randomly selected slides re-reading by a second lab technologist.

### Anemia diagnosis

Anemia was diagnosed via laboratory confirmatory method using hemoglobin level and classification according to the WHO. Normal hemoglobin concentration corresponded to a value between 12 mg/dl and above, and mild between 10.0–10.9 mg/dl, moderate 7.0–9.9 mg/dl and severe less than 7.0 mg/dl. The hemoglobin level was conducted for each pregnant woman at the enrollment and during delivery.

### Ethical considerations

Before enrollment participants were informed about consent process based on aspects of rights, respects, benefits, compensation, confidentiality, withdrawal and voluntarily participation. Written informed consent was signed by each participant before the study begins. For each illiterate participant written informed consent was obtained from a parent and/or legal guardian. Ethical approval was sought from the Ethics Review Committee of Mount Kenya University, and a research permit obtained from NACOSTI (MKU/ERC/2100, license No. NACOSTI/P/22/16233), as well as local authorizations from County and Webuye hospital management.

### Sample calculation and data analysis

The sample size calculation formula for cohort was used based on the prevalence of malaria in the non-exposed group, which was estimated at 28% according to the study of Nyamu [16]. The prevalence of malaria in the exposed group estimated at 6.1% according to the DHIS2 [11]. Beta (10%), Alpha (5%), Confidence level of 95%, Z alpha (1.96), Z beta value (1.28), Sample size for group-1(n1 = 60), Sample size for each group (n1 = 60), Sample size for both group (n1 + n2 = 120), Attrition (=20%), Total sample size with attrition = 144.

Afterward, data were fed cleaned, interpreted, edited and coded into SPSS 27 version, Chi-square and Fisher’s Exact were computed for categorical data, and relative risk was used for low birth weight which was the outcome of interest. The confidence level Alpha or error term used in this study was 0.05 (95%).

### Study design

**Fig. 1:**
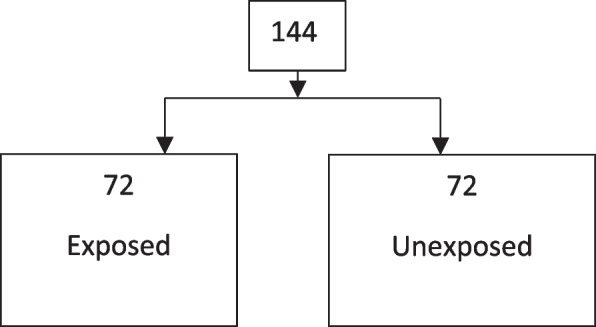
Study design

A prospective cohort study was expected to enroll 144 participants after malaria test and to arrange them in two arms; exposed and non-exposed. Each group expected 72 participants (Fig. 1).

### Socio-demographic characteristics of pregnant women

There was strict observance in provision of consent by the participants, and malaria test done after taking blood samples. Malaria test was conducted via microscopy in order to confirm the diagnosis. A total of 140 (100%) were tested for malaria of which 70 (50%) tested positive for malaria and 70 (50%) tested negative with 3% of loss to follow up. Overall, the majority were in the 18–25 years’ age-group, primigravida, married, with secondary level of education, middle income level, self-employed, residents of rural areas and in the second trimester. There was significant association between marital status (*p*-value < 0.001), gestation in weeks (p-value < 0.001) and area of residence (*p*-value < 0.028) had a weak association (Table 1).

**Table 1:** Socio-demographic characteristics of pregnant women

Variables		Malaria test		
	Overall *N* = 140	Negative *n* = 70	Positive *n* = 70	*p*-value
**Age in years, n (%)**				0.220
18–25	89 (63.6)	40 (57.1)	49 (70.0)	
26–33	36 (25.7)	20 (28.6)	16 (22.9)	
34–41	15 (10.7)	10 (14.3)	5 (7.1)	
**Parity, n (%)**				0.190
Primigravida	53 (37.9)	22 (31.4)	31 (44.3)	
Secondgravida	40 (28.6)	20 (28.6)	20 (28.6)	
Multigravida	32 (22.9)	21 (30.0)	11 (15.7)	
Grand multigravida	15 (10.7)	7 (10.0)	8 (11.4)	
**Marital status, n (%)**				< 0.001
Married	117 (83.6)	67 (95.7)	50 (71.4)	
Single	23 (16.4)	3 (4.3)	20 (28.6)	
**Level of education, n (%)**				0.21
None/Primary	38 (27.1)	15 (21.4)	23 (32.9)	
Secondary	67 (47.9)	34 (48.6)	33 (47.1)	
College/University	35 (25.0)	21 (30.0)	14 (20.0)	
**Occupation, n (%)**				0.369
Employed	20 (14.3)	11 (15.7)	9 (12.9)	
Unemployed	50 (35.7)	21 (30.0)	29 (41.4)	
Self-employed	70 (50.0)	38 (54.3)	32 (45.7)	
**Income earning, n (%)**				0.056
Low	49 (35.0)	18 (25.7)	31 (44.3)	
Middle	55 (39.3)	33 (47.1)	22 (31.4)	
High	36 (25.7)	19 (27.1)	17 (24.3)	
**Area of residence, n (%)**				0.028
Rural	73 (52.1)	30 (42.9)	43 (61.4)	
Urban	67 (47.9)	40 (57.1)	27 (38.6)	
**Gestational age, n (%)**				< 0.001
First trimester	43 (30.7)	33 (47.1)	10 (14.3)	
Second trimester/Third trimester	97 (69.3)	37 (52.9)	60 (85.7)	

Socio-demographic characteristics of participants were computed using Chi-square test and Fisher’s Exact *p*-value < 0.05 (95%)

### Characteristics of newborns

The prevalence of low birth weight was 4.6% either 6 cases. Normal birth weight were 124/140 cases, 63/70 (95.4%) were in malaria negative cohort and 61/70 (95.3%) in malaria positive cohort. There were 140 pregnancies of whom 129 (92.2%) were live births, only 4 (2.29) were admitted, 1 (0.7%) stillbirth in the cohort of positive malaria, 10 (7.1%) were miscarriages. Very low birth weight represented 1case (0.8%) in positive malaria pregnant women and low birth weight had 5 (3.8%), of whom 3 (4.7%) observed in positive malaria cohort and 2 (3.0%) in negative malaria cohort. Normal deliveries were 115 (88.5%), caesarian section 15 (11.5%). Females 78 (60%) against male newborns 52 (40%). There was no statistical difference with regard to birth weight in both groups (*p*-value = 0.790). Therefore, newborns characteristics were not statistically significant in both positive and negative malaria cohorts (Table 2).

**Table 2:** Characteristics of newborns

Variables		Malaria test		
	Overall, *N* = 140	Negative, *n* = 70	Positive, *n* = 70	*p*-value
**Conception product outcome, n (%)**				0.820
Miscarriage	10 (7.1)	4 (5.7)	6 (8.6)	
Stillbirth	1 (0.70)	0 (0.00)	1 (1.4)	
Alive	125 (89.3)	64 (91.4)	61 (87.1)	
Admitted	4 (2.9)	2 (2.9)	2 (2.9)	
**Birthweight for single baby, n (%)**				0.790
Very low	1 (0.8)	1 (1.5)	0 (.00)	
Low	5 (3.8)	2 (3.0)	3 (4.7)	
Normal	119 (91.5)	61 (92.4)	58 (90.6)	
Macrosomia	5 (3.8)	2 (3.0)	3 (4.7)	
**Mode of delivery, n (%)**				0.450
Normal	115 (88.5)	57 (86.4)	58 (90.6)	
CS	15 (11.5)	9 (13.6)	6 (9.4)	
**Sex for single child, n (%)**				0.830
Males	52 (40.0)	27 (40.9)	25 (39.1)	
Females	78 (60.0)	39 (59.1)	39 (60.9)	

Conception product outcome, mode of delivery and sex of the child were computed at a *p*-value < 0.05

### Associated conditions versus birth weight among pregnant women

Fisher’s exact were used to determine the association between birth weight versus anemia, hypertensive disease, diabetes and HIV. The results show that most newborns who had normal birth weight were from non-anemic women 89 (68.5%) at *p*-value 0.923, were HIV negative 125 (96.2) *p*-value > 0.99, were normotensive 125 (96.2) *p*-value > 0.99, were non-diabetic 128 (98.5) *p*-value > 0.99. HIV was diagnosed in malaria positive women 4 (5.7%) versus 1 (1.4%) in malaria negative with a total prevalence rate of 3.8%. Strikingly, relative risk was calculated for anemia (RR: 0.996, 95% C.I:0.917–1.081), and malaria test (RR:0.999, 95% CI:0.926–1.077). A relative risk less than 1 means that there was no statistical difference between the two groups (Table 3).

**Table 3:** Associated Conditions versus birth weight among pregnant women. *N* = 140

Associated diseases		Birth weight			
	Overall, *N* = 130	Abnormal, *n* = 6	Normal, *n* = 124	Fisher’s exact *p*-value	RR 95%CI
**Anaemia, n (%)**				0.923	
Normal	89 (68.5)	4 (66.7)	85 (68.5)		
Abnormal	41 (31.5)	2 (33.3)	39 (31.5)		0.996 (0.917–1.081)
**HIV infection, n (%)**				> 0.99	
Non-reactive	125 (96.2)	6 (100.0)	119 (96.0)		
Reactive	5 (3.8)	0 (0.0)	5 (4.0)		
**Hypertension in pregnancy, n (%)**				> 0.99	
Normal	125 (96.2)	6 (100.0)	119 (96.0)		
High	5 (3.8)	0 (0.0)	5 (4.0)		
**Gestational diabetes, n (%)**				> 0.99	
Normal	128 (98.5)	6 (100.0)	122 (98.4)		
Gestational diabetes	2 (1.5)	0 (0.0)	2 (1.6)		
**Malaria test results, n (%)**				> 0.969	0.999 (0.926–1.077)
Negative	66 (50.8)	3 (50.0)	63 (50.8)		
Positive	64 (49.2)	3 (50.0)	61 (49.2)		

Relative risk equal to 1 means that there is no risk, above 1 means that the risk exists,and below one is protective

### Associated conditions during pregnancy by malaria result (microscopy)

After computing Chi-square of association between malaria results versus anemic pregnant women, gestational hypertensive, HIV and gestational diabetes, the results show that there was statistical association between malaria results and anemia (*p*-value < 0.001). Otherwise, there was no statistical difference between malaria results and HIV (*p*-value = 0.370), hypertension (*p*-value = 0.058), and gestational diabetes (*p*-value > 0.5). There were 2 positive malaria cases (2.9%) with severe malaria. The portion of pregnant women with HIV reactive results were higher among the positive malaria cohort as compare to negative cohort with 4 (5.7%) versus 1 (1.4%) respectively. Consequently, it was worth noting that among negative malaria cohort women there were 5 (7.1%) pre-eclamptic cases and 2 (2.9%) gestational diabetes. Among the malaria positive cohort there was no case of pre-eclampsia nor gestational diabetes (Table 4).

**Table 4:** Associated conditions during pregnancy by malaria results

Variables	Overall, *N* = 140	Malaria test		*p*-value
		Negative, *n* = 70	Positive, *n* = 70	
**Anaemia, n (%)**				< 0.001
Normal	96 (68.6)	61 (87.1)	35 (50.0)	
Mild	30 (21.4)	9 (12.9)	21 (30.0)	
Moderate	12 (8.6)	0 (0.0)	12 (17.1)	
Severe	2 (1.4)	0 (0.0)	2 (2.9)	
**HIV infection, n (%)**				0.370
Non-reactive	135 (96.4)	69 (98.6)	66 (94.3)	
Reactive	5 (3.6)	1 (1.4)	4 (5.7)	
**Hypertension in pregnancy, n (%)**				0.058
Normal	135 (96.4)	65 (92.9)	70 (100.0)	
High	5 (3.6)	5 (7.1)	0 (0.0)	
**Gestational diabetes, n (%)**				0.500
Normal	138 (98.6)	68 (97.1)	70 (100.0)	
Gestational diabetes	2 (1.4)	2 (2.9)	0 (0.0)	

Anaemia was categorized as mild 10–12, moderate 8–10 and severe < 7 g/dl, hypertension or pre-eclampsia 140/90 mmHg, gestational diabetes mellitus when blood glucose was above 7.8 mmol/l during oral glucose tolerance test

## Discussion

The prevalence of low birth weight due to malaria was estimated at 4.6% in our study. This prevalence seems low as compare to the study conducted in Nigeria by Iwuchukwu and Vincent [17] who found 26%, Cates et al. [18] found that the risk of delivering low birth weight was estimated at 8.8%. The low birth weight observed in our study could be due to the low sample size and also the reason that our study was conducted at one-site. Marital status was found to be strongly associated with malaria in our study (*p*-value< 0.001). The majority of pregnant women were married. Similar findings were demonstrated in the Democratic Republic of Congo [8], Cameroon [19], Kenya [20] where studies reported that the husband support was very effective for malaria prevention during pregnancy. In Yaounde [21] single pregnant women had 4-fold time risk of getting malaria than married pregnant women. The role of marital status was demonstrated as very determinant in healthcare seeking behavior [22] demonstrated that it contributed to improve economic status of pregnant women, marriage can also improve economic growth of the family [23]. Married pregnant women had less likelihood to contract malaria which can improve pregnancy outcomes, whereas single pregnant women may lack social support to attend early antenatal clinic and lack access to the preventive measure which can contribute to negative pregnancy outcomes.

Gestational age was associated with malaria in pregnancy at a *p*-value < 0.001. The majority of pregnant women were in the second and third trimester of pregnancy. Similarly, the study conducted in Ethiopia by Gontie et al. [24] found that the second trimester had higher odds of developing malaria. Tuike et al. [25] found that the first trimester was associated with high risk of malaria in primigravida. Women can be also infected in all trimesters Nosten et al. [26]. Importantly, the increased risk in pregnant women was established in primigravida due to the lack of acquired immunity [27, 28]. Hounkonnou et al. 2020 [29] found high risk of association with low birth weight in early microscopic pregnancy among primigravida. In Malawi, Grote et al. 2010 [30] found that there was a high risk of low birth weight in the second trimester. Thompson et al. 2020, [31] found that there was 63% of risk associated with low birth weight among pregnant women with malaria during delivery. In Malawi, Kalilani et al. [32] found that there was a high risk of low birth weight in the second trimester.

The area of residence influenced the level of transmission and the severity of malaria with the notion of pre-acquired antimalarial immunity, previous exposure to the infection and the acquired immunity [33]. In Uganda, the urban set up was associated with reduction of mosquito density [34]. In Guinea, residence was a risk factor for both peripheral and placental malaria [35]. Low birth weight as an important marker of newborn death or survival [36, 37]. In our study, pregnant women were enrolled from 16 weeks’ gestation based on exposure to malaria [positive cohort] and non-exposed [negative cohort] followed until delivery to determine the birth weight. We found that the presence of malaria in pregnancy was not significantly associated with birth weight. Comparing pregnant women with malaria and without malaria [RR = 0.999, Fisher’s Exact > 0.969] and low birth weight (*p*-value 0.790). There was no difference in birth weight in both cohorts at delivery. Even the outcome of interest which was the low birth did not differ. The presence of malaria alone or with other illnesses in birth cohorts, did not result in significant negative birth outcomes as similarly corroborated in research carried out in Tanzania and Sudan [38, 39]. At current, as per Ministry of health and WHO directives all pregnant women in malaria endemic zones are encouraged to attend antenatal clinic and at the same time receive standards of care and this could have influenced the current trend of malaria as demonstrated by Bhatt et al. 2015 [40]. The pathway from malaria to low birth weight is well-known through placental triggering mechanism via expression to VAR2CSA, the unique surface antigen responsible of sequestration of *Plasmodium falciparum* evolving in the process of inflammation with vasogenesis, angiogenesis and nutrient transportation dysregulation which affect the fetal development [41]. The ealier intervention can curb negative effect of malaria before initiating prevention measures which was found associated with low birth weight Heng [42]. Use of cot effective interventions were beneficial to reduce the burden of low birth weight like in Malawi, the evident benefit of independent or the combined use of both intermittent preventive treatment and long lasting insecticide treated nets was found significantly associated with 20% low birth weight decrease Nkoka [43]. Two or more doses of intermittent preventive treatment were associated with the reduction of low birth weight while one dose of IPT/SP was not associated with the reduction Kayentao [44]. Studies highlighted on quality interventions were as an alternative to prevent malaria negative outcomes such maternal folic acid supplementation in low- and middle-income countries, which was found associated with an increased mean birthweight and decreases in the incidence of low birthweight and small for gestational age, while exposure to more than 4 antenatal visits decreased incidence of low birth weight [45–49]. Iron and folic acid supplementation was associated with increased birth weight in studies conducted in low-middle income countries [50, 51]. In our study we found that 89% of participants with malaria were treated with Artemether-lumefantrine. The Use of Artemether-lumefantrine was associated with trend towards decreased low birth weight and pregnancy loss. Although, current evidence suggesting that the efficacy of antimalarial drugs in preventing low birth weight may decrease with *Plasmodium* resistance, antimalarial medications were used for prevention during pregnancy and showed a significant low birth weight reduction of 27% in the cohort that used the drug when compared with the control group [52]. Malnutrition and malaria share the same geographical area and it contributes to increased disease burden in pregnancy. However, both appear important contributors to low birth weight, and nutrient supplementation during pregnancy appear to be an attractive and feasible intervention to minimize the risk of low birth weight [53]. In Kenya and Congo Democratic Republic, it was established that the association between malaria infection and reduced fetal growth was greatest among malnourished women. However, in Benin, the effect of malaria infection on fetal growth velocity was greatest among women with low anthropometric status [54, 55]. A Kenyan national survey showed that 2 years after Covid-19 pandemic there was an increase of the ANC four visit from 48 to 66%, 88% live births occurred in the health facility and 89% of delivery were assisted by a skilled provider. In the meantime, there was a significant decrease of home delivery from 34% in 2009 to 11% in 2022 [56].

## Conclusion

We observed in this study that the presence of malaria did not result in reduced birth weight. Identifying risk factors associated with malaria in endemic zones will be beneficial for targeting priority interventions. The study findings may be due mainly to the concurrent interventions such as antenatal care, public health policy implementation, socioeconomic factors, malaria case management, nutritional status of pregnant women, which have been emphasized on during the last two decades. Therefore, implementing malaria cost-effective strategies in the current context will contribute to spearhead good maternal and child health outcomes.

### Limitations

The findings of this research study should be interpreted with caution since the study was carried out at one-site and targeting pregnant women who attended antenatal clinic in the hospital set up. Pregnant women and their babies born at home were not included.

### Recommendation

Further multiple site longitudinal studies are needed to be carried out in different malaria prone zones including home delivery to determine the effect of each intervention in the context of malaria and low birth weight.

## Data Availability

Data included in this study are openly and directly accessible within the manuscript and if any other material not displayed here is needed, it can be obtained upon formal and written request addressed to the main author.

## Abbreviations

ANC: Antenatal Clinic
C/S: Caesarian Section
CIPD: County Integrated Development Plan
HIV: Human Immunodeficiency Virus
KNBS: Kenya National Bureau of Statistics
KSH: Kenyan Shilling
RR: Relative Risk
SPSS: Statistical Package for Social Science
WHO: World Health Organization
